# Sandwiching Phosphorene with Iron Porphyrin Monolayer for High Stability and Its Biomimetic Sensor to Sensitively Detect Living Cell Released NO

**DOI:** 10.1002/advs.202104066

**Published:** 2022-01-02

**Authors:** Chunmei Zhang, Fangxin Hu, Xijuan Hao, Qianghai Rao, Tao Hu, Wei Sun, Chunxian Guo, Chang Ming Li

**Affiliations:** ^1^ Institute of Materials Science and Devices School of Materials Science and Engineering Suzhou University of Science and Technology Kerui Road Suzhou 215009 P. R. China; ^2^ College of Chemistry and Chemical Engineering Hainan Normal University Haikou 571158 P. R. China

**Keywords:** 2D materials, biomimetic catalyst, monolayer assembly, phosphorene, stability

## Abstract

Instability of 2D phosphorene material is the major obstacle for its broad applications. Herein phosphorene is sandwiched with self‐assembled iron porphyrin monolayers on both sides (I‐Phene) to significantly enhance stability. Iron porphyrin has strong interaction with phosphorene through formation of P—Fe bonds. The sandwich structure offers excellent stability of phosphorene by both‐sided monolayer protections for an intact phosphorene structure more than 40 days under ambient conditions. Meanwhile, the electron transfer between iron porphyrin and phosphorene result in a high oxidation state of Fe, making I‐Phene biomimetic sensitivity toward oxidation of nitric oxide (NO) for 2.5 and 4.0 times higher than phosphorene and iron‐porphyrin alone, respectively. Moreover, I‐Phene exhibits excellent selectivity, a wide detection range, and a low detection limit at a low oxidation potential of 0.82 V, which is comparable with the reported noble metal based biomimetic sensors while ranking the best among all non‐noble biomimetic ones. I‐Phene is further used for real‐time monitoring NO released from cells. This work provides effective approach against phosphorene degrading for outstanding stability, which has universal significance for its various important applications, and holds a great promise for a highly sensitive biomimetic sensor in live‐cell assays.

## Graphical abstract

A sandwich structure of phosphorene assembled with iron porphyrin exhibits enhanced stability under ambient conditions which overcomes drawback of phosphorene. The sandwiched material acts as a biomimetic catalyst for high‐performance NO sensing, realizing in situ monitoring NO in live‐cell assays.

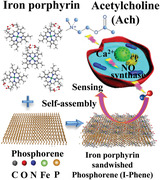

## 1 Introduction

Since its first discovery by exfoliation in 2014,^[^ ^1^ ^]^ thin black phosphorus (BP) nanosheet namely phosphorene has exhibited much potential applications in multifarious fields such as the optical conductors,^[^ ^2^ ^]^ photothermal therapy,^[^ ^3^ ^]^ solar water splitting,^[^ ^4^ ^]^ field effect transistors,^[^ ^5^ ^]^ rechargeable batteries,^[^ ^6^ ^]^ hydrogen generation,^[^ ^7^ ^]^ and photocatalytic reaction.^[^ ^8^ ^]^ However, phosphorene still battles with its poor stability resulting from easy degradation by water and oxygen.^[^ ^9^ ^]^ The roles of oxygen and water playing in BP degradation have been studied by Huang et al.,^[^ ^10^ ^]^ revealing that the degradation is initiated by oxygen. Since the lone pair electrons of P in BP can easily react with oxygen to form P*_x_* O*_y_*, which can be removed by water to further expose P° to continue oxidation. To enhance BP stability, many methods have been developed. The use of organic solvents is the most direct approach to keep away from oxygen and water, and the solvation shell can protect the nanosheets from reaction with air and water for BP stability.^[^ ^11^ ^]^ However, this method makes BP only usable in organic solutions, thus limiting its applications. Surface modification has been widely employed to protect BP.^[^ ^12^ ^]^ BP‐based materials can be modified with azidobenzoic acid,^[^ ^10b^ ^]^ Ag^+^,^[^ ^13^ ^]^ CaP,^[^ ^14^ ^]^ Au@glutathione, and Fe_3_O_4_@polyetherimide.^[^ ^15^ ^]^ BP has also been coated by TiO_2_,^[^ ^16^ ^]^ Al_2_O_3_,^[^ ^17^ ^]^ MoS_2,_^[^ ^18^ ^]^ and Co nanoparticles.^[^ ^19^ ^]^ These modifications can enhance the stability of BP in certain degree but always severely affect some important properties of BP, eventually leading to restricted applications. It is thus crucial to explore more effective ways for protection of BP while avoiding negative effects of thick layer modifications on its important applications.

Biomimetic “enzymes” as artificial analogues of natural enzymes have gained increasing attention in various applications such as cascade reactions,^[^ ^20^ ^]^ hydrosilylation reaction,^[^ ^21^ ^]^ and lithium–sulfur batteries,^[^ ^22^ ^]^ because they can accelerate the rate of specific chemical reactions with high activity and specificity. However, phosphorene‐based biomimetic sensing material has not been investigated yet because of its poor stability especially in solutions. Thus, a delicate design to fabricate biomimetic phosphorene‐based material with a novel structure for high stability is essential in its biomimetic applications.

Although the lone‐pair electrons of phosphorene can result in instability to hurt its practical applications, they can serve as electron donor sources to accelerate the interfacial electron transfer for excellent catalysis.^[^ ^23^ ^]^ Wang et al.^[^ ^23^ ^]^ and Li et al.^[^ ^24^ ^]^ investigated Pt and PtRu metal nanoparticles deposited on phosphorene and found that phosphorene can manipulate the electronic structures through metal‐P bonds toward water splitting. However, these Pt and PtRu nanoparticles are not able to produce an intact monolayer to full cover both sides of phosphorene with complete protection for good stability.

Porphyrins possessing 2D molecular structure without central metals have been chemically covalently linked to various nanomaterials including carbon nanotubes,^[^ ^23^ ^]^ graphene,^[^ ^24^ ^]^ MoS_2_,^[^ ^25^ ^]^ and phosphorene^[^ ^26^ ^]^ through amide bonds, direct C‐S linkage, and P—C bonds, respectively. These works are focused on the bonds related to carbon. Only recently one published paper reports theoretical calculation (first‐principles) results of metalloporphyrins‐phosphorene hybrid nanostructures involving metal‐P interaction (metal: Fe, Co, Ni, and Zn).^[^ ^27^ ^]^ Metal‐P coordination has been reported.^[^ ^28^, ^29^ ^]^ However, how to use metal‐P interaction between metal porphyrin and phosphorene to construct unique sandwich structure of monolayers of porphyrins on both sides of phosphorene as well as the effects on its stability and catalytic properties have never been investigated.

In this work, for the first time we experimentally self‐assemble monolayers on both side of phosphorene for a sandwich structure, in which the formation of P—Fe bonds offers a full protection to significantly improve the stability of phosphorene while making a higher oxidation state of Fe as more active oxidation catalysis centers toward more sensitive detection of NO. In addition, the I‐Phene sensor is used to biomimetically in situ real time monitor NO released from living cells. This work provides effective approach against phosphorene degrading for outstanding stability, which has universal significance for a stabilized phosphorene in various important applications.

## 2 Results and Discussion

**Figure 1:**
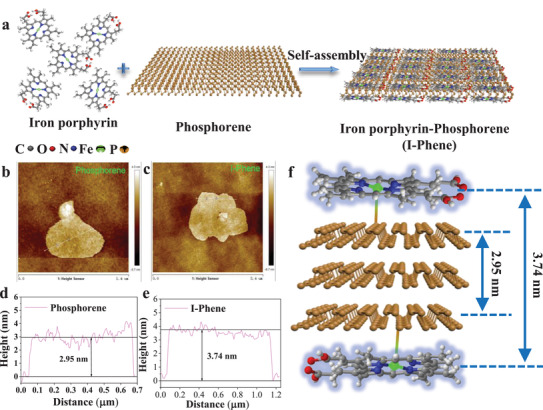
a) Schematic illustration of iron porphyrin and phosphorene and the formation of stable monolayer‐sandwiched phosphorene I‐Phene. AFM images of b) phosphorene, c) I‐Phene together with the corresponding height profiles of d) phosphorene and e) I‐Phene. f) Structure configuration of the I‐Phene.

As schematically shown in **Figure** **1**a, iron porphyrin monolayer is self‐assembled on phosphorene in IPA solution. First, experimental results indicate that I‐Phene is easier to be centrifuged from IPA solution (Figure S1a, Supporting Information), suggesting that iron porphyrin and phosphorene have strong interaction. The UV–vis spectroscopic studies were used to investigate the interaction of phosphorene and iron porphyrin and further formation of I‐Phene (Figure S1b, Supporting Information). Iron porphyrin (hemin) exhibits a strong peak at 400 nm attributing to the Soret band and a group of weak peaks between 500 and 700 nm ascribing to the Q‐bands.^[^ ^30^ ^]^ Phosphorene shows the absorption of BP crosses the UV and entire visible light region because of its unique electronic structure.^[^ ^15^ ^]^ Remarkably, I‐Phene exhibits relatively stronger absorption than that of phosphorene in all the absorbance range of 300–800 nm, and also the characteristic adsorption peaks of iron porphyrin, which prove the successfully assembly of iron porphyrin on phosphorene to form the I‐Phene. In addition, photoluminescence measurements were performed as shown in Figure S1c, Supporting Information. Notably, a strong peak around 1.6 eV is found for phosphorene. The thinner layers of BP were easily destroyed by laser used in photoluminescence tests, resulting in an enhanced intensity at the bandgap of 1.6 eV.^[^ ^31^ ^]^ While I‐Phene and iron porphyrin are featureless because of iron porphyrin protection, resulting in less affection by laser.

To gain insight into the layer and crystal structure of I‐Phene, atomic force microscopic (AFM) and X‐ray diffraction (XRD) measurements were conducted. Figure 1b–e and Figure S1d–g, Supporting Information show AFM images and height profiles of phosphorene and I‐Phene. The AFM measurements for each thickness were performed by 30 times, and the measured average thicknesses of phosphorene and I‐Phene are 2.95 and 3.74 nm, respectively. Since the reported average thickness of monolayer phosphorene from literatures is ≈0.85 nm,^[^ ^32^ ^]^ the phosphorene used in our experiments should be three layers. Further, the thickness of I‐Phene (3.74 nm) is larger than 3‐layer phosphorene by 0.79 nm, which should be the thickness of the modified metal‐porphyrin layer. The thickness of iron porphyrin calculated by Materials Studio is 0.35 nm (Figure S1h, Supporting Information), indicating iron porphyrin is two layers. Based on first‐principles study,^[^ ^27^ ^]^ the top adsorption conformation of iron porphyrin on one side of phosphorene forming P—Fe bond with the lowest adsorption energy has the most stability. Therefore, the two layers of iron porphyrin should be on both side of phosphorene for a stable sandwich structure as schematically shown in Figure 1f. XRD results display that the I‐Phene has a relatively weaker peak than those of phosphorene (Figure S2a, Supporting Information), which could be resulted from the surficial iron porphyrin monolayer coverage on phosphorene. There is no additional XRD signal for iron porphyrin (Figure S2b, Supporting Information) in the I‐Phene, which indicates no crystal formation for the iron porphyrin in the I‐Phene but only surface immobilization, further confirming the formation of iron porphyrin monolayer on the I‐Phene.

**Figure 2:**
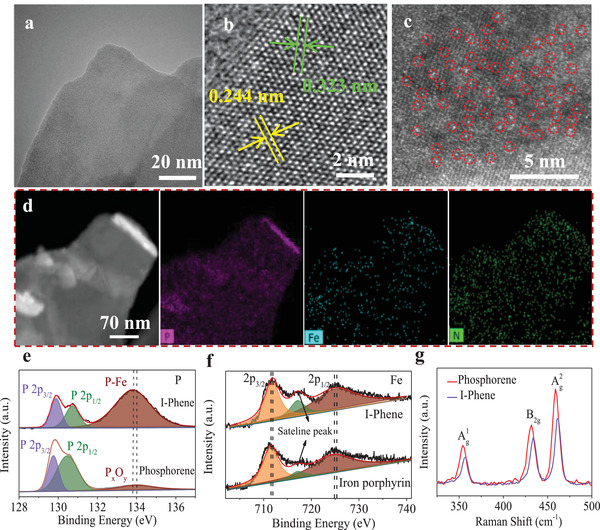
a,b) HRTEM images of I‐Phene at different magnifications; c) AC HAADF‐STEM images of I‐Phene, bright spots in red circles are Fe single atoms; d) HAADF‐STEM image of I‐Phene and the corresponding elemental mappings of P, Fe, and N; high‐resolution XPS survey of e) P 2p in phosphorene and I‐Phene and f) Fe 2p in iron porphyrin and I‐Phene. g) Raman spectra of phosphorene and I‐Phene.

Morphology and structure of I‐Phene were characterized by transmission electron microscopy (TEM), aberration‐corrected high‐annular dark‐field scanning TEM (AC HAADF‐STEM), and SEM. The phosphorene used in this work has a nanosheet structure (Figure S3a, Supporting Information) with a crystal lattice of 0.244 nm (Figure S3d, Supporting Information) due to the matrix of few‐layered BP sheets,^[^ ^33^ ^]^ which agrees with the AFM results. After sandwiching with iron porphyrin, the I‐Phene retains the nanosheet structure, as shown in **Figure** **2**a and Figure S4a, Supporting Information. Figure 2b displays that I‐Phene has lattice fingers of 0.244 and 0.323 nm that corresponds to (014) and (012) of BP, respectively, informing that the assembly of iron porphyrin does not affect the crystal structure of phosphorene.^[^ ^34^ ^]^ No Fe lattice diffraction is found in I‐Phene, suggesting that iron porphyrin is in a form of molecule distribution with monolayer structure rather than clusters. The Fe content in I‐Phene is 2.13% (At%). The molecular distribution of iron porphyrin in the I‐Phene is further confirmed by AC HAADF‐STEM (Figure 2c). It clearly exhibits quite uniform distribution of bright spots in red circles that represent Fe atoms on I‐Phene surface without overlap. In addition, the fast Fourier transform (FFT) can be used to identify the BP thickness. According to theoretical and experimental results,^[^ ^31^, ^35^ ^]^ the intensity ratio of (110) to (200) diffraction peaks (*I*_110_/*I*_200_) for a thinner BP sheet is greater than one for monolayer and smaller than one for multilayer. The FFT patterns comparison of phosphorene (Figure S3e, Supporting Information) and I‐Phene (Figure S4b, Supporting Information) indicates that I‐Phene is composed of thinner BP nanosheets.^[^ ^36^ ^]^ HAADF‐STEM image and the corresponding elemental mappings (Figure 2d; Figure S4c–e, Supporting Information) of I‐Phene show that C, Fe, N, and O are uniformly dispersed on phosphorene sheet suggesting a successful self‐assembly of iron porphyrin on phosphorene. Similarly, SEM results (Figures S5,S6, Supporting Information) prove the successful preparation of I‐Phene. In brief, although phosphorene was covered by the iron porphyrin layer, iron porphyrin did not alter the structure and physical properties of phosphorene.

To further study the interaction and electron transfer between phosphorene and iron porphyrin, X‐ray photoelectron spectroscopy (XPS) was conducted (Figure 2e,f; Figure S7, Supporting Information). The high‐resolution N 1s, C 1s, and O 1s spectra of I‐Phene, phosphorene, and iron porphyrin (Figure S7, Supporting Information) demonstrate that iron porphyrin is successfully self‐assembled on the phosphorene. Obviously, I‐Phene displays a strong peak at 133.7 eV in Figure 2e, corresponding to P‐Fe, which is different from the P*_x_* O*_y_* peak at 134 eV in phosphorene.^[^ ^37^ ^]^ The 0.3 eV negative shift of I‐Phene belongs to the interaction of P and Fe, indicating electron density of P increases.^[^ ^38^ ^]^ In contrast, after modification Fe 2p peaks shift to a higher binding energies with 0.5 eV increase for reduced electron density (Figure 2f). These results are very consistent with the theory of XPS analysis, in which an enhanced binding energy indicates a reduced electron density, while a reduced binding energy suggests an increased electron density.^[^ ^19^, ^33^, ^39^ ^]^ Therefore, we can reasonably argue that the lower and higher binding energy shifts of P 2p and Fe 2p in I‐Phene are attributed to the increased and decreased electron density of phosphorene and I‐Phene, respectively, which symbols electrons transfer from Fe to P through the iron porphyrin/phosphorene interaction resulting in higher oxidation state of Fe.

Raman spectroscopy was performed to further characterize the phosphorene and I‐Phene (Figure 2g). Both phosphorene and I‐Phene exhibit three peaks, corresponding to the out‐plane vibrational mode A1 g, in‐plane vibrational mode B2 g, and in‐plane vibrational mode A2 g, respectively. Obviously, the Raman intensities of all three peaks were decreased after the self‐assembly of iron porphyrin. The decreased intensities are very likely to be caused by the disruption of intralayer phosphorus bonding after the self‐assembly processing.^[^ ^26^, ^40^ ^]^ Furthermore, all three peaks of I‐Phene show clearly blue shift with 3 cm^−1^, which are ascribed to the electron transfer between iron porphyrin/phosphorene interface.^[^ ^18^ ^]^ In particular, the oscillation of P atoms is also enhanced to some extent when iron porphyrin is self‐assembled on the phosphorene surface, which increases the corresponding Raman scattering energy and produces blue‐shifts.^[^ ^28^ ^]^ In addition, according to previous studies,^[^ ^34^, ^41^ ^]^ the three peaks of BP shift to higher wavenumber with the decreased layers. The blue shift could be partly due to the thin layer of phosphorene in I‐Phene, indicating iron porphyrin can avoid the agglomeration of phosphorene.

**Figure 3:**
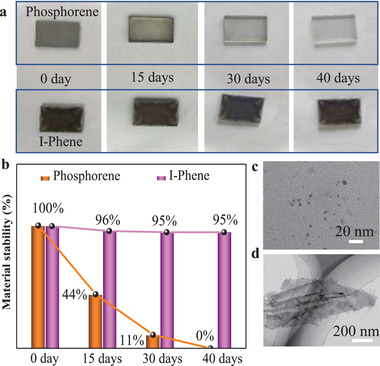
a) Pictures of phosphorene and I‐Phene modified on the glass slice taken on the beginning, 15 days, 30 days, and 40 days in air. b) The corresponding stability change diagrams for phosphorene and I‐Phene in (a). c) HRTEM image of phosphorene and d) TEM image of I‐Phene obtained from samples stored in IPA after 1 month.

The instability of phosphorene in air is of paramount concern for its practical applications. To evaluate the influence of immobilization of iron porphyrin on phosphorene stability, we exposed phosphorene and I‐Phene in air and recorded their change (**Figure** **3**). Since instability of phosphorene resulted in color change from brown to colorless in exposure to air, Figure 3b was obtained by measuring the transparency degree to evaluate the decay of phosphorene. Pictures of phosphorene and I‐Phene exposed in air for different days (Figure 3a; Figure S8, Supporting Information) and the corresponding stability comparison (Figure 3b) reveal that phosphorene sheets were gradually corroded to colorless and no phosphorene left after 40 days, while I‐Phene sheets behaved no obvious change with 95% left, indicating that iron porphyrin self‐assembled on both sides of phosphorene effectively protected phosphorene from oxidation. In contrast, phosphorene alone was oxidized even stored in IPA solvent. As seen in Figure 3c,d, after storage in IPA for 1 month, phosphorene changed to quantum dots (QD), while I‐Phene sheets had no obvious change. Nanosheet structure can be transformed into QD because of its reaction with oxygen and photo‐assisted oxidation reaction resulting in the yields structural decay of phosphorene.^[^ ^9^ ^b,^ ^10^ ^b]^ In addition, the extent of oxidation for phosphorene both on glass slide and in IPA solution were measured by XPS (Figure S8c, Supporting Information). Initially, only 17.56% of phosphorene in IPA was oxidated, however most phosphorene was oxidated once dropped on glass slide and the surface oxidation extent was up to 71.9%. After 40 days, 43.78% of phosphorene was left in IPA solution, and phosphorene on the glass slide was completely transformed into PO_4_^3−^. In contrast, for I‐Phene, since the surface was modified by iron porphyrin, resulting in P bonded with Fe, initially only P‐Fe was observed. After 40 days, also no P‐O observed in Figure S8d, Supporting Information, suggesting I‐Phene was stable.

The as‐prepared stable I‐Phene is further used to electrochemically detect NO in aqueous solution. NO is not only atmospherically toxic and anaerobic ammonium oxidation dependent molecule,^[^ ^42^ ^]^ but also a key reactive species in organisms, which plays an indispensable role in many physiological processes containing immune response regulation, participation in vasodilation, blood pressure regulation, and production of inflammatory mediators.^[^ ^43^ ^]^ NO is also associated with tumor carcinogenesis.^[^ ^44^ ^]^ Thus, monitoring of NO released from live cells is essential for pathology and medicinal research. However, the detection of NO in biological systems is still a challenge, due to its short half‐life, trace level released from cells, and rapid diffusion and easily oxidated by O_2_.^[^ ^45^ ^]^ The reversible redox pair of Fe (III)/Fe (II) in iron porphyrin can behave as a catalytic center toward oxidation of NO.^[^ ^46^ ^]^ It is expected that stronger oxidation state of Fe in I‐Phene can deliver faster electrooxidation reaction at the Fe (III)/Fe (II) catalysis centers toward NO oxidation.

**Figure 4:**
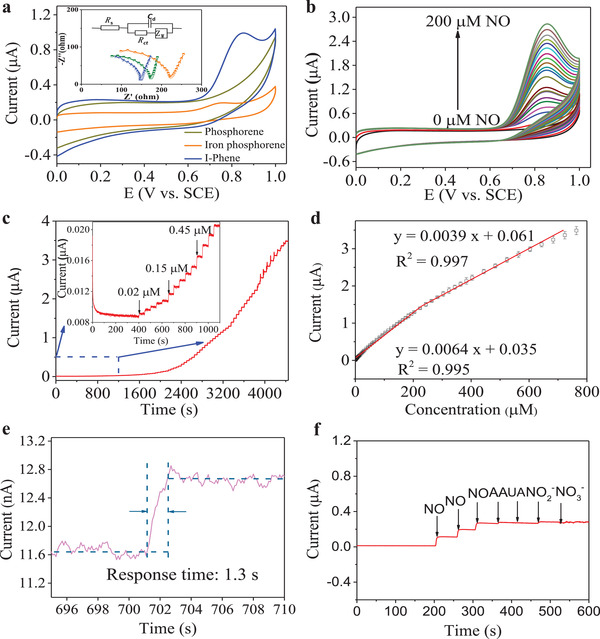
a) Cyclic voltammograms (CVs) of phosphorene, iron porphyrin, and I‐Phene in 0.1 m PBS with 50 µm NO, scan rate 20 mV s^−1^; inset is the EIS of different materials. b) CVs of I‐Phene in 0.1 m PBS with different concentrations of NO from 0 to 200 µm, scan rate 20 mV s^−1^. c) Amperometric response of I‐Phene to the successive addition of different concentrations of NO in 0.1 m PBS at the potential of 0.82 V. The inset in (c) is the amplified response to lower concentrations of NO. d) Calibration curves of the I‐Phene to NO. e) Response time of I‐Phene toward NO. f) Amperometric response of I‐Phene to 10 uM NO, AA, UA, NO_2_^−^, and NO_3_^−^.

The electrochemical properties of I‐Phene were studied by different electrochemical testing techniques. The I‐Phene clearly exhibits higher peak oxidation current in the PBS toward NO than that of phosphorene and iron‐porphyrin by ≈2.5 and 4.0 times, respectively (**Figure** **4**a; Figure S9a, Supporting Information). Apparently, I‐Phene showed onset NO oxidation potential at 0.6 V and NO oxidation peak at 0.82 V (Figure 4a). In order to investigate the interfacial electron transfer properties of the sensitive materials toward NO, electrochemical impedance spectroscopy (EIS) tests were carried out. The measured Nyquist plots of phosphorene, iron porphyrin, and I‐Phene in PBS with NO (inset, Figure 4a) shows defined semicircles. It is known that the interfacial charge transfer resistance (*R*_ct_) is equal to the diameter of the semicircle, and thus a smaller semicircle indicates a higher interfacial charge transfer rate.^[^ ^47^ ^]^ The calculated *R*_ct_s for phosphorene, iron porphyrin, and I‐Phene are 167.2, 211.8, and 136.9 Ω, respectively, clearly confirming that high‐oxidized Fe on the sandwiched I‐Phene can accelerate the NO oxidation for better sensing.

Figure 4b displays the CV curves of I‐Phene toward different concentrations of NO. Without NO in electrolyte, no response could be observed. With the successive addition of 10 µm NO, obvious oxidation currents can be seen and increasing along with the increased NO concentration. Figure S9b, Supporting Information displays the relationship between oxidation peak currents and the concentrations of added NO. A good linearity was obtained with a linear regression equation of *I* (µA) = 0.028 *C*_NO_ (µm) + 0.0255 (*R* ^2^ = 0.997). Similarly, DPV (Figure S10a, Supporting Information) was also used to examine the I‐Phene sensing performance toward NO. The corresponding linear curve is illustrated in Figure S10b, Supporting Information and the linear regression equation is *I* (µA) = 0.0058 *C*_NO_ (µm) + 0.48 (*R* ^2^ = 0.997).

To further investigate the sensing performance of the I‐Phene for NO detection, the *i–t* responses were recorded when successively adding NO (Figure 4c). A ladder‐like plot was obtained with the successive addition of NO and an obvious increase was observed even toward 0.02 µm NO (Figure 4c, inset). Figure 4d displays calibration plots for I‐Phene with two linear ranges, 0.02–243.65 µm and 243.65–683.65 µm, respectively. The calculated limit of detection (LOD) is 6 nm (S/N of 3). The NO detection potential of I‐Phene is lower than the previously reported materials listed in Table S1, Supporting Information, especially non precious metals, such as Cu_2_O@FePO_4_^[^ ^48^ ^]^ and N‐G/FePc/Nafion/PLL ITO.^[^ ^45^ ^]^ It can be noted that the detection potential for materials in Table S1, Supporting Information are comparable with I‐Phene in Table S1, Supporting Information, but they are precious metal such as gold and platinum, all of which suffer from high expense and low specificity. Furthermore, the linear range of I‐Phene is much wider than other reported materials and the LOD is lower than most reported materials in Table S1, Supporting Information.

It is noted that I‐Phene can rapidly reach a steady‐state toward NO with a fast response time of 1.3 s (Figure 4e), beating down the short NO half‐life of 3–6 s for real‐time detections of living cells. The anti‐interference ability of the I‐Phene for detecting NO was also studied through *i–t* curve by adding ascorbic acid (AA), uric acid (UA), NO_2_^−^, and NO_3_^−^ in PBS containing NO. As shown in Figure 4f, the I‐Phene display a remarkable current response for NO, but no significant signal when injecting AA, UA, NO_2_^−^, and NO_3_^−^, suggesting an excellent anti‐interference ability and selectivity of I‐Phene. The electrochemical stability of I‐Phene was also estimated (Figure S11, Supporting Information), indicating I‐Phene was stable in PBS.

**Figure 5:**
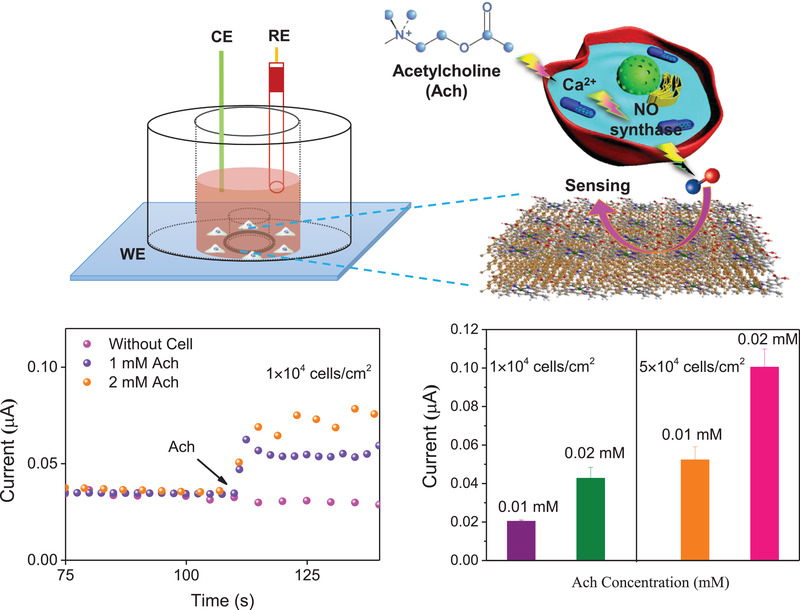
I‐Phene based electrochemical sensor for real‐time NO monitoring. Illustration for real‐time in situ NO detection in cervical cancer cells (Hela) (1 × 10^4^ and 5×10^4^ cells cm^−2^) stimulated by different concentrations of Ach (0.01 and 0.02 mm).

I‐Phene further demonstrated its application in in situ real‐time measurement of NO molecules released from living cervical cancer cells (Hela). It was recorded by testing *i–t* response of I‐Phene toward NO oxidation (**Figure** **5**). Acetylcholine (Ach) has been reported to be an efficient stimulus for living cells to generate NO via activating NO synthase (NOS), while hemoglobin (Hb) as a kind of NOS inhibitor can effectively inhibit the generation of NO.^[^ ^49^ ^]^ Ach can cause an influx of Ca^2+^ to bind the receptors on the surface of Hela cells and the increased Ca^2+^ activated endothelial NO synthase to generate NO.^[^ ^50^ ^]^ By contrast, Hb can consume and scavenge NO efficiently.^[^ ^49c^ ^]^ Once a same amount of Ach (0.01 and 0.02 mm, Figure 5; Figure S12, Supporting Information) were added into different amount of Hela cells (1 × 10^4^ and 5 × 10^4^; Figure S12a,b, Supporting Information), the current increased obviously. The stronger current response of 0.02 mm than that of 0.01 mm reveals that NO release process is drug‐concentration dependent. In addition, the larger current responses of 5 × 10^4^ samples than those of 1 × 10^4^ sample (Figure S12c,d, Supporting Information) reveal that the NO release is cell‐concentration dependent process. When a same amounts of Ach and Hb (0.02 and 0.01 mm; Figure S12e,f, Supporting Information) were injected, no obvious current increase was observed, indicating that the generation of NO can be inhibited effectively. These results demonstrate that I‐Phene can be used for real‐time detecting NO molecules released from cells in live‐cell assays.

## 3 Conclusion

In summary, we present a delicate design and for the first time to experimentally self‐assemble iron porphyrin monolayer on both side of phosphorene for highly stable sandwich‐structured I‐Phene. It is found that phosphorene and iron porphyrin have strong interfacial interaction to form P—Fe bonds. Due to the sandwich structure with monolayer protection, the I‐Phene well retains its intact structure for more than 40 days under ambient conditions for excellent stability while plain phosphorene degrades completely. The electron transfer between iron porphyrin and phosphorene leads to a high oxidation state of Fe, which results strong electrooxidation catalysis toward highly sensitive electrochemical detection of NO, delivering sensitivity much higher than phosphorene and ironporphyrin alone by 2.5 and 4.0 times, respectively. Moreover, I‐Phene achieves a wide detection range of 0.02–683.65 µm and a low detection limit of 6 nm at a low oxidation potential of 0.82 V, which is advantageous over many other sensing materials. I‐Phene is further applied in monitoring NO released from living cells. This study presents a delicate design to effectively protect phosphorene from degrading, while the disclosed scientific insights behind have universal significance to guide unique synthesis of stable phosphorene for various important applications. As a demonstration of stable phosphorene application, this work holds a great promise for sensitive biomimetic NO sensors in practical clinic diagnosis in real‐time live‐cell assays.

## 4 Experimental Section

The experiments and methods are described in the Supporting Information.

## Conflict of Interest

The authors declare no conflict of interest.

## Supporting information

Supporting Information

## Data Availability

Research data are not shared.
