# Supplementary material for: Sandwiching Phosphorene with Iron Porphyrin Monolayer for High Stability and Its Biomimetic Sensor to Sensitively Detect Living Cell Released NO
Source: Adv Sci (Weinh). 2022 Jan 2;9(6):2104066. doi: 10.1002/advs.202104066 (PMC8867151; doi:10.1002/advs.202104066)
Supplement: Supplementary file 1 — Supporting Information [file ADVS-9-2104066-s001.pdf]

ADVANCED SCIENCE
Open Access

Supporting Information

for *Adv. Sci.*, DOI: 10.1002/advs.202104066

Sandwiching Phosphorene with Iron Porphyrin Monolayer for High Stability and Its Biomimetic Sensor to Sensitively Detect Living Cell Released NO

Chunmei Zhang, Fangxin Hu, Xijuan Hao, Qianghai Rao, Tao Hu, Wei Sun, Chunxian Guo, Chang Ming Li **

Dr. C. Zhang, Dr. F. Hu, X. Hao, Q. Rao, Dr. T. Hu, Prof. C. Guo, Prof. C. M. Li
Institute of Materials Science and Devices, School of Materials Science and Engineering, Suzhou University of Science and Technology, Kerui Road, Suzhou 215009, P.R. China
E-mail: cxguo@usts.edu.cn

Prof. W. Sun, College of Chemistry and Chemical Engineering, Hainan Normal University, Haikou 571158, P R China

Prof. C. M. Li
Institute for Clean Energy & Advanced Materials, Faculty of Materials and Energy, Southwest University, Chongqing 400715, P.R. China
E-mail: ecmli@swu.edu.cn

Experimental Section

Chemicals and reagents: Hemin ($C_{34}H_{32}ClFeN_4O_4$, ≥95%) was acquired from Sigma-Aldrich. Isopropyl alcohol (IPA, C_3H_8O, ≥99.7%) was purchased from Jiangsu Qiangsheng Functional Chemistry Co., Ltd. Phosphorene was customized from Nanjing Xianfeng Nanomaterials

Supporting Information

Sandwiching Phosphorene with Iron Porphyrin Monolayer for High Stability and Its Biomimetic Sensor to Sensitively Detect Living Cell Released NO

Chunmei Zhang, Fangxin Hu, Xijuan Hao, Qianghai Rao, Tao Hu, Wei Sun, Chunxian Guo, Chang Ming Li **

Dr. C. Zhang, Dr. F. Hu, X. Hao, Q. Rao, Dr. T. Hu, Prof. C. Guo, Prof. C. M. Li
Institute of Materials Science and Devices, School of Materials Science and Engineering, Suzhou University of Science and Technology, Kerui Road, Suzhou 215009, P.R. China
E-mail: cxguo@usts.edu.cn

Prof. W. Sun, College of Chemistry and Chemical Engineering, Hainan Normal University, Haikou 571158, P R China

Prof. C. M. Li
Institute for Clean Energy & Advanced Materials, Faculty of Materials and Energy, Southwest University, Chongqing 400715, P.R. China
E-mail: ecmli@swu.edu.cn

Experimental Section

Chemicals and reagents: Hemin ($C_{34}H_{32}ClFeN_4O_4$, ≥95%) was acquired from Sigma-Aldrich. Isopropyl alcohol (IPA, C_3H_8O, ≥99.7%) was purchased from Jiangsu Qiangsheng Functional Chemistry Co., Ltd. Phosphorene was customized from Nanjing Xianfeng Nanomaterials Technology Co., Ltd with a low concentration of 0.2 mg mL^{-1} (1-10 layers), which was prepared by liquid phase exfoliation in a week. Before the use, phosphorene was further sonicated in an ice bath for at least half an hour. Phosphate buffer solution (PBS, 0.1 mol L^{-1}, pH 7.4) was prepared using NaH_2PO_4 and Na_2HPO_4. All chemicals used were at least analytical reagent grade, and the solutions were prepared by ultrapure water (18.25 MΩ/cm).

Synthesis of sandwiched iron porphyrin monolayer-sandwiched phosphorene (I-Phene): I-Phene was prepared by the self-assembly process without adding any surfactants. 1 mL Phosphorene in IPA (0.2 mg mL^{-1}) was added into 6 mL hemin dispersed in IPA under nitrogen

protection. Afterward, the mixture was stirred for 48 h under a nitrogen atmosphere. After that, the suspension was centrifuged at 10000 rpm for 10 min to obtain the final product.

Preparation of nitric oxide (NO) saturated PBS: NO saturated PBS solution was prepared in a fume hood according to the previous reported study.[1] Briefly, 2 M H_2SO_4 was cautiously added dropwise into saturated $NaNO_2$ solution in a round bottom flask under the condition of continuous stirring. The produced NO was passed through saturated KOH, 10% (w/v) KOH and 2.5% (w/v) KOH, respectively, to remove other nitrogen oxides produced from the reaction. Finally, NO was bubbled in 0.1 M PBS solution until to reach saturation state (1.8 mM). The NO saturated PBS solution should be stored in a nitrogen-protected environment and was kept at 4 ^{o}C for further use.

Material characterizations: UV-vis spectra were obtained from a UV-2600 spectrophotometer. Transmission electron microscopy (TEM) and high resolution TEM (HRTEM) images were obtained from FEI Talos F200x (TEM). The aberration-corrected scanning transmission electron microscopy (AC-STEM) images were obtained on FEI Titan Themis 60-300. X-ray powder diffraction (XRD) patterns were collected on a Bruker D8 Advance X-ray diffractometer using a Cu Kα radiation source scanning from 10° to 60° with a rate of 5° min^{-1}. Atomic force microscopy (AFM) was performed on a Bruker Dimension Icon AFM, and the thickness measured was based on 30 statistics. X-ray photoelectron spectroscopy (XPS) tests were conducted on a VG Thermo ESCALAB 250 spectrometer operated at 120 W. The binding energies were all calibrated by the carbon 1 s at 284.6 eV. All characterizations in this study should be conducted as soon as possible, once samples were prepared.

Fabrication of electrochemical sensor and electrochemical measurements: Before electrode modification with materials, glassy carbon (GC) electrodes were polished using alumina powder (0.3 and 0.05 μm in diameter) followed by washing and sonication to obtain a mirror surface. The phosphorene and I-Phene were modified on GC for further use.

All electrochemical measurements were conducted on a CHI 660E electrochemical workstation with a standard three-electrode system. The sample modified GC electrode, an Ag/AgCl electrode and a platinum wire were used as the working, reference and counter electrodes, respectively. Before the electrochemical measurements, the PBS solution should be first degassed by N_2 to remove O_2. 0.1 M (pH = 7.4) phosphate buffer solution (PBS) prepared by NaH_2PO_4 and Na_2HPO_4 was used as the electrolyte in the electrochemical experiments. Cyclic voltammograms (CVs) were tested in the potential range from 0 to 1.0 V at the san rate of 20 mV s^{-1} in N_2 saturated PBS solution. Differential pulse voltammetry (DPV) was performed in the potential range from 0.4 to 1.0 V. Amperometric measurements (i-t curves) were tested in N_2 saturated PBS (0.1 M, pH = 7.4) solution under stirring.

Cell culture and in situ detection of NO: Human cervical cancer cells (Hela**)** was obtained from Rayme Biotechnology Co., Ltd. Hela were supplemented with 10% fetal bovine serum and 1% antibiotic and cultured in a humidified incubator (95% air with 5% CO_2) at 37 ^{o}C with complete culture medium. Real-time detection of NO released from living cells was measured by chronoamperometry. Hemoglobin (Hb) as a kind of NO-synthase inhibitor can inhibit the generation of NO effectively.

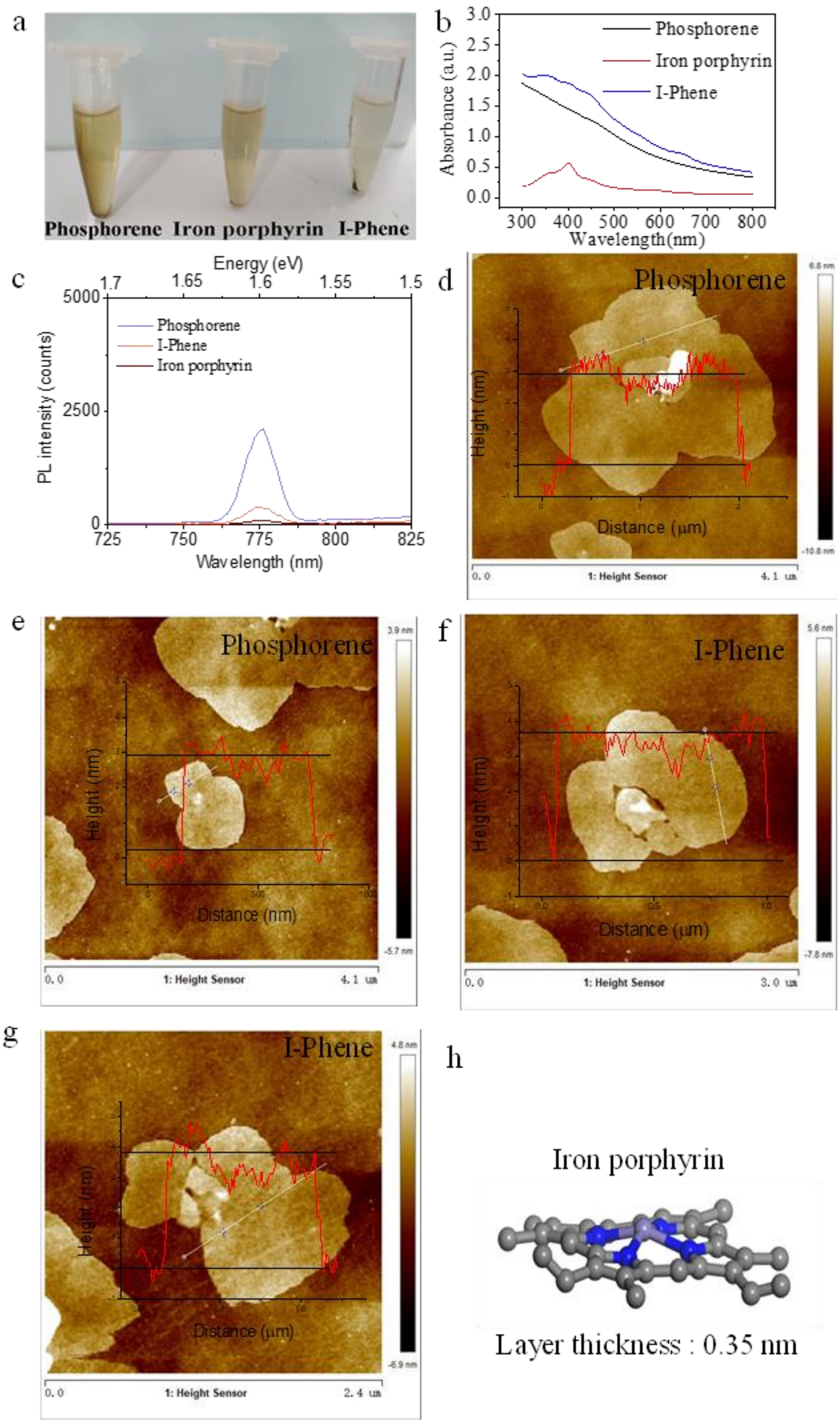

z

Figure S1. a) The pictures of phosphorene, iron porphyrin and I-Phene after centrifugation at 10000 rpm for 10 min. b) UV-visible spectra of phosphorene, iron porphyrin and I-Phene in IPA solution. c) Photoluminescence spectra acquired on phosphorene, I-Phene and iron

porphyrin. AFM images of phosphorene d-e) and I-Phene f-g). Thickness of iron porphyrin calculated by Materials Studio h).

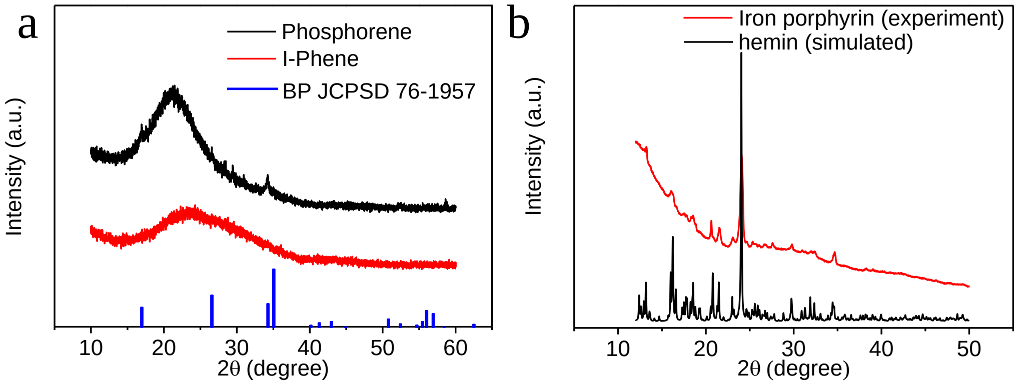

Figure S2. XRD pattern of a) phosphorene, I-Phene and the standard pattern (blue) of BP in JCPDS NO. 76-1957 and b) iron porphyrin (experiment) and hemin (simulated).

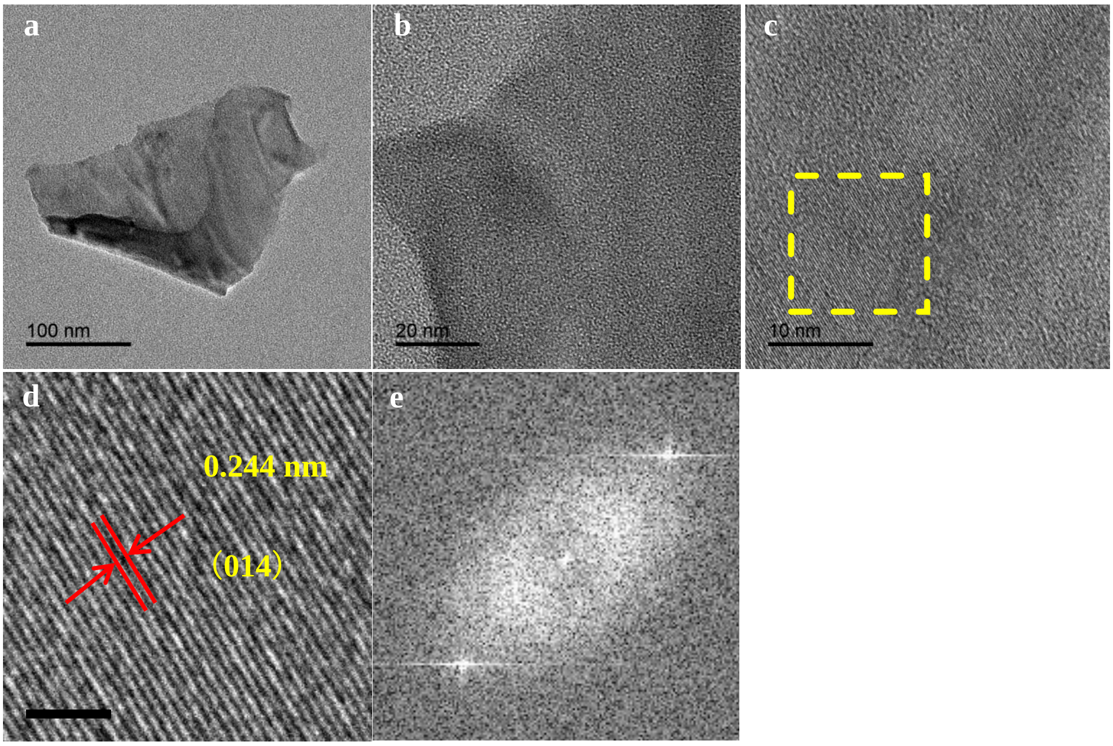

Figure S3. a) TEM and b-d) HRTEM images of phosphorene. d) is the enlarge image of c) in yellow square. e) is the FFT of c) in yellow square.

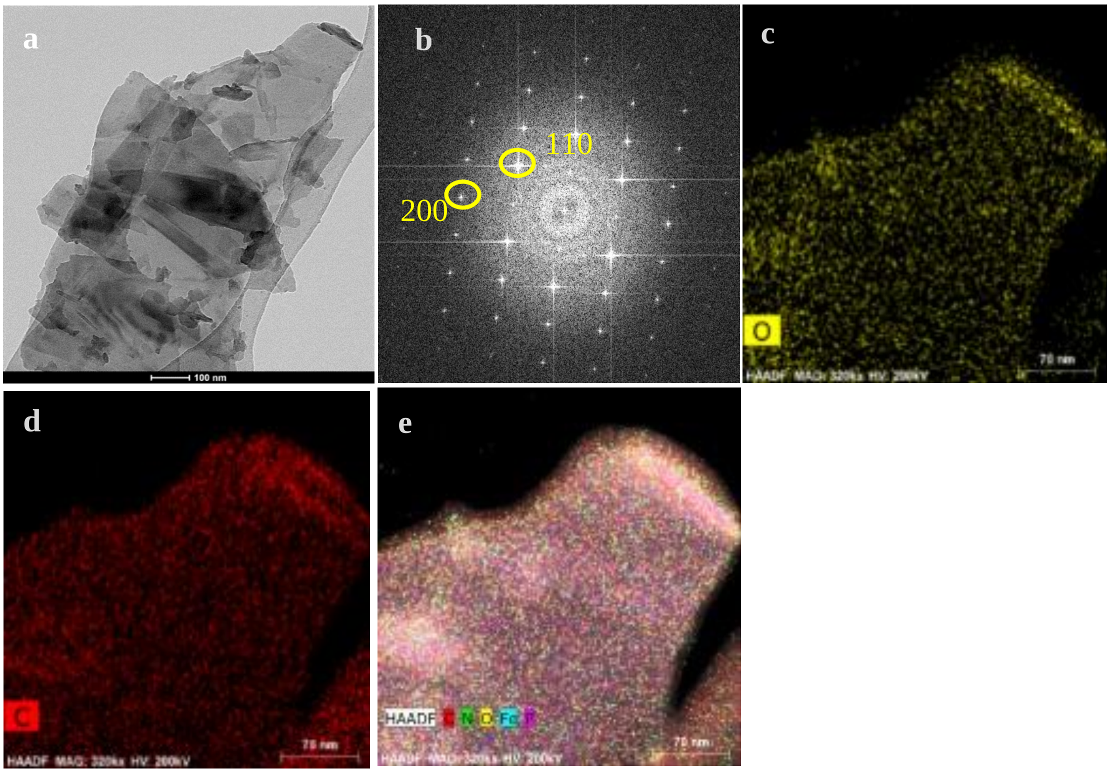

Figure S4. a) TEM, b) the corresponding FFT images of I-Phene, c-e) the corresponding elemental mappings of O, C and the overlap image of all elements for I-Phene, scale bars are 70 nm.

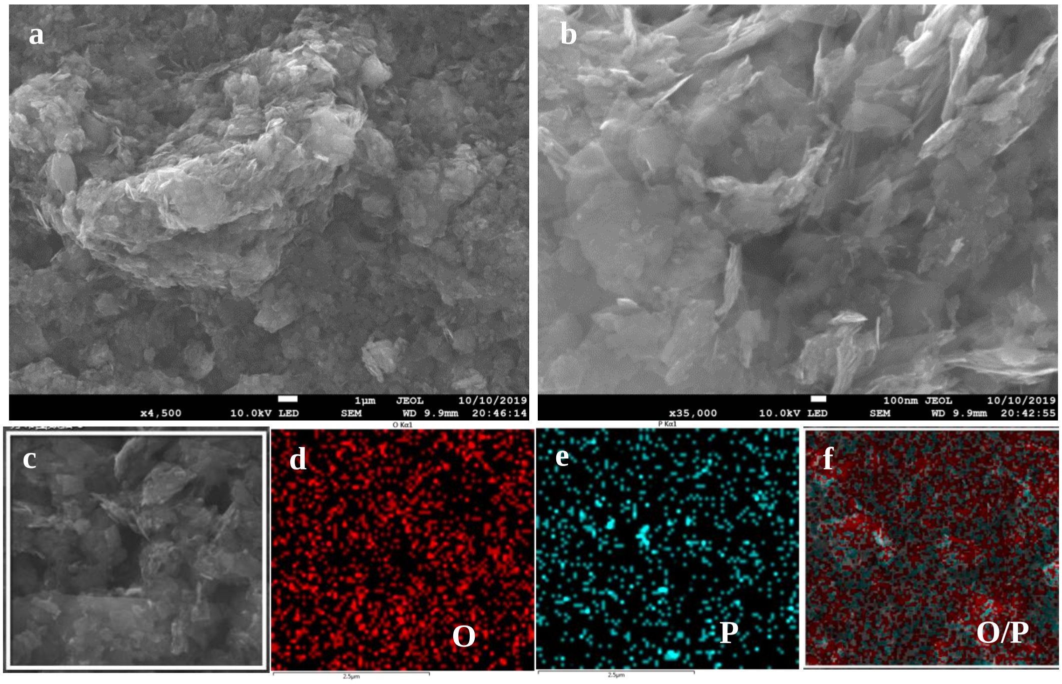

Figure S5. a-b) SEM images at different magnification of phosphorene. c-f) Elemental mappings.

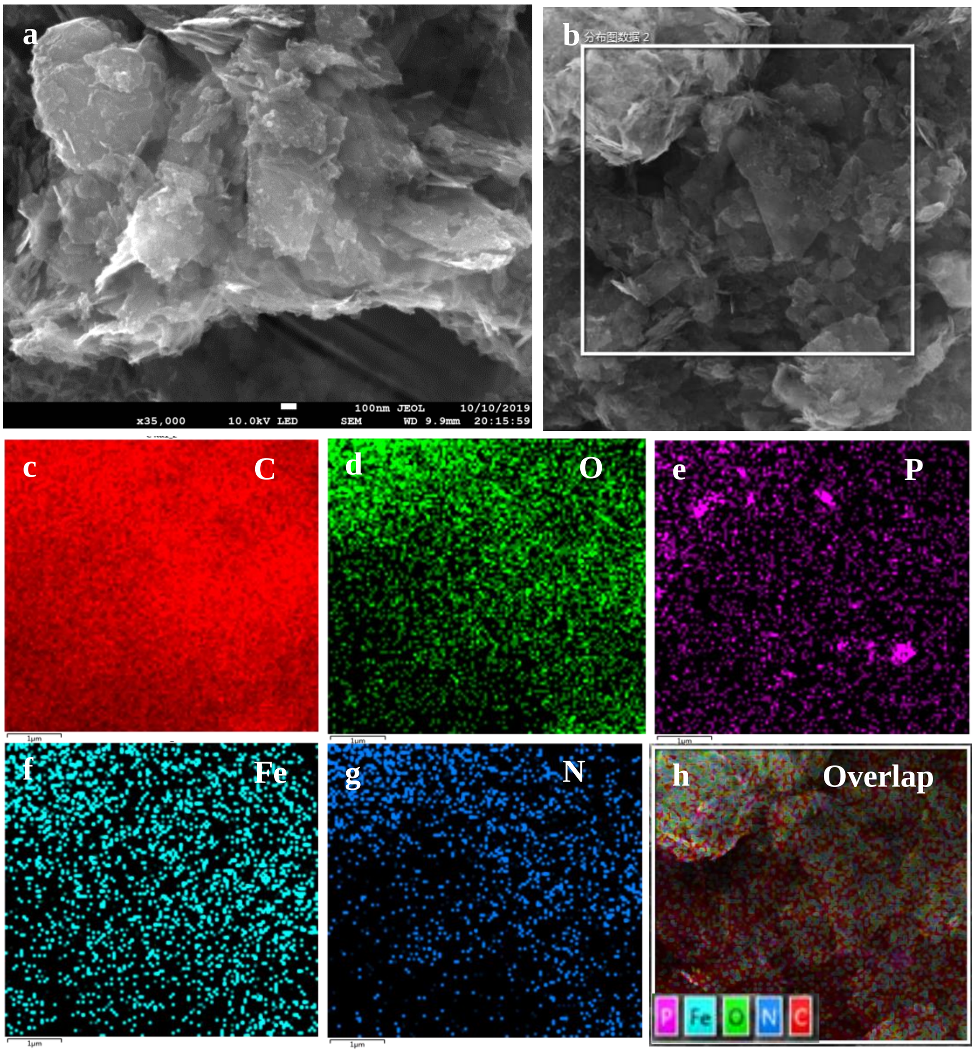

Figure S6. a) SEM image of I-Phene. b-h) Elemental mappings.

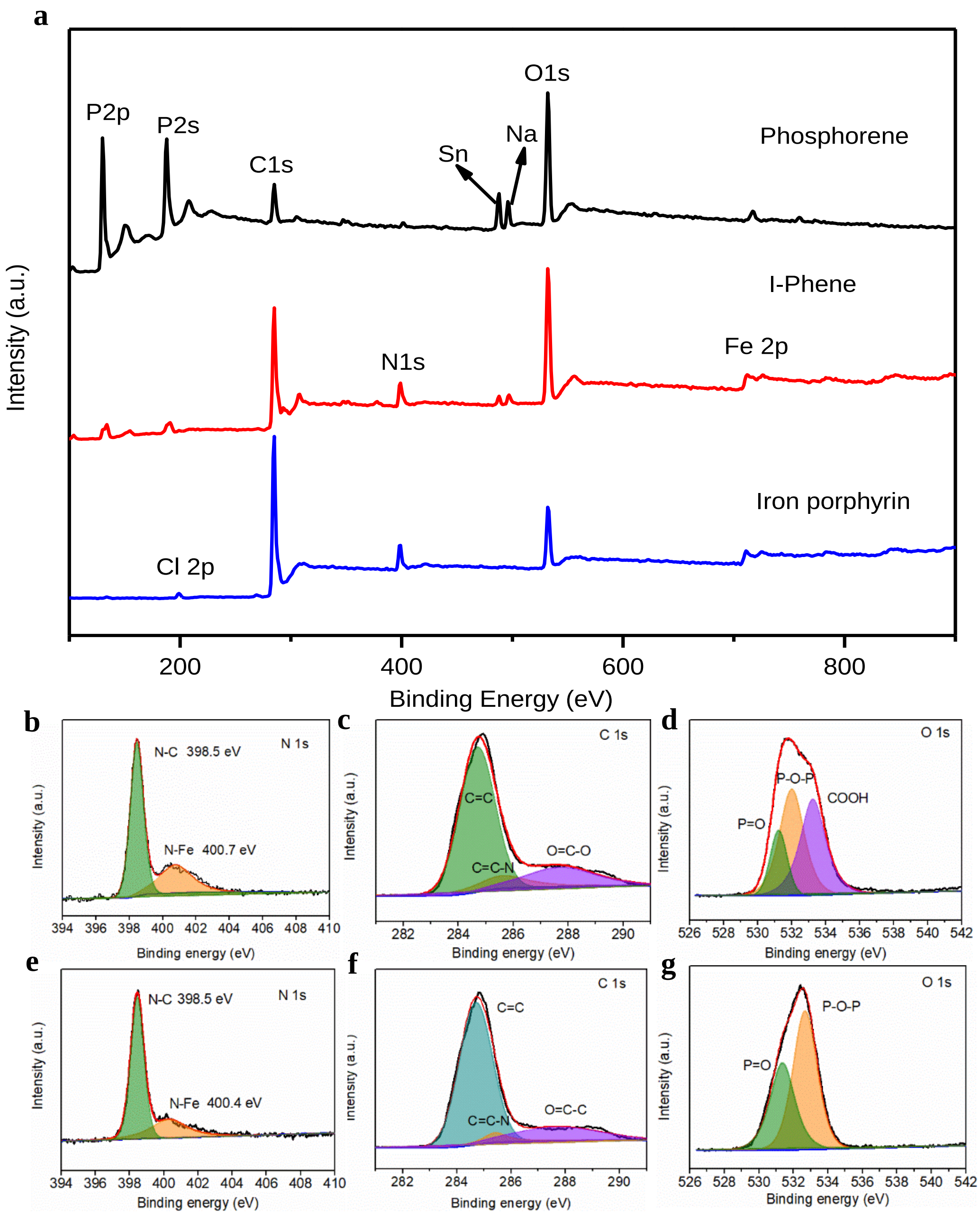

Figure S7. a) XPS survey spectra of phosphorene, I-Phene and iron porphyrin. High-resolution XPS survey of b) N 1s and c) C 1s and d) O 1s in I-Phene, e) N 1s and f) C 1s in iron porphyrin, g) O 1s in phosphorene.

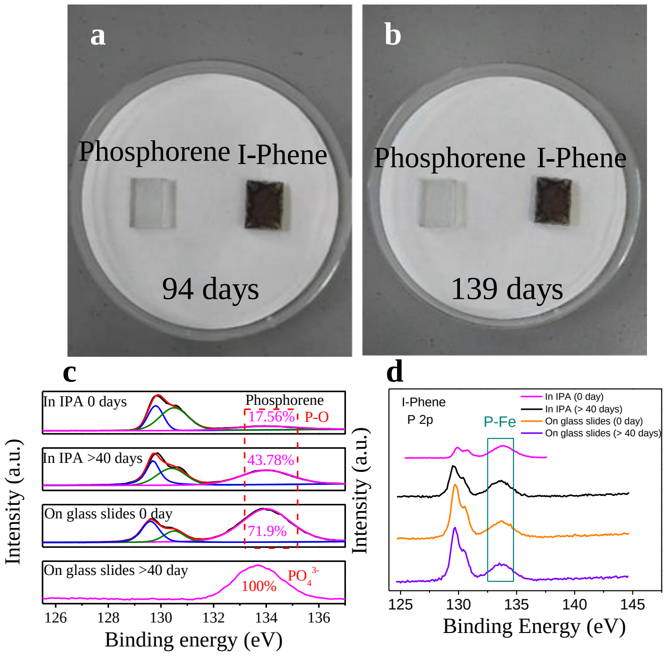

Figure S8. Pictures of phosphorene and I-Phene exposed in air after a) 94 days and b) 139 days. P 2p XPS surveys of phosphorene c) and I-Phene d) stored in IPA and on glass slides for different days.

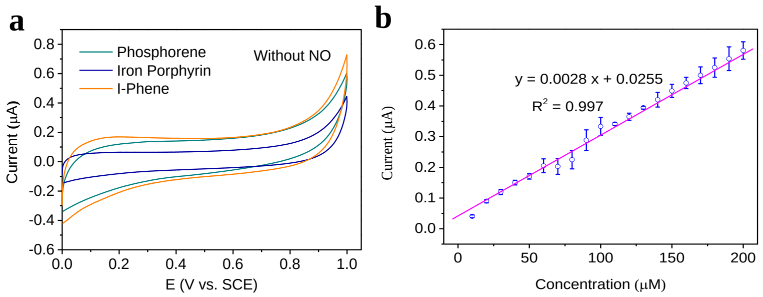

Figure S9. a) CV of f phosphorene, iron porphyrin and I-Phene in N_2-saturated 0.1 M PBS without NO. b) Corresponding calibration curve of I-Phene for NO detection by CV measurements.

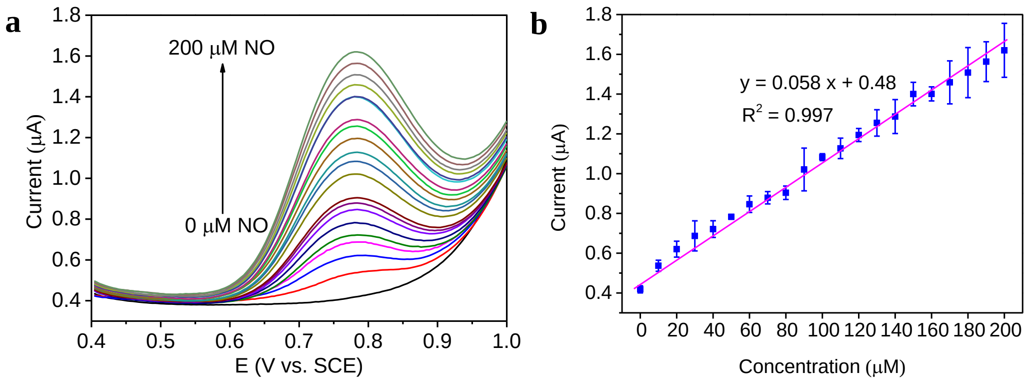

Figure S10. a) DPV of I-Phene to different concentrations of NO. b) Calibration curves of the I-Phene to NO.

Table S1. Comparison for different materials of the previous studies and this work.

Materials	Detection potential (V)	Linear range (μM)	LOD (μM)	References
$Cu_2O@FePO_4$ core-cage	0.97	1.35-337.0 nM<br>1.6-13.5 μM	0.00045	[2]
N-G/FePc/Nafion/PLL ITO	0.9	0.18-400 μM	0.18	[3]
Au/PDMS	0.85	0.01-1.295	0.001	[4]
Au NPs-3DGH	0.81	0.2-6	0.009	[5]
Graphene-gold	0.8	10-5000	0.04	[6]
rGO-Ag nanocomposite	0.96	10-220	2.84	[7]
rGO-Co_3O_4@Pt	0.84	10-650	1.73	[8]
rGO–CeO_2	-	0.018 -5.6	0.0096	[9]
I-Phene	0.82	0.02-243.65<br>243.65-683.65	0.006	This work

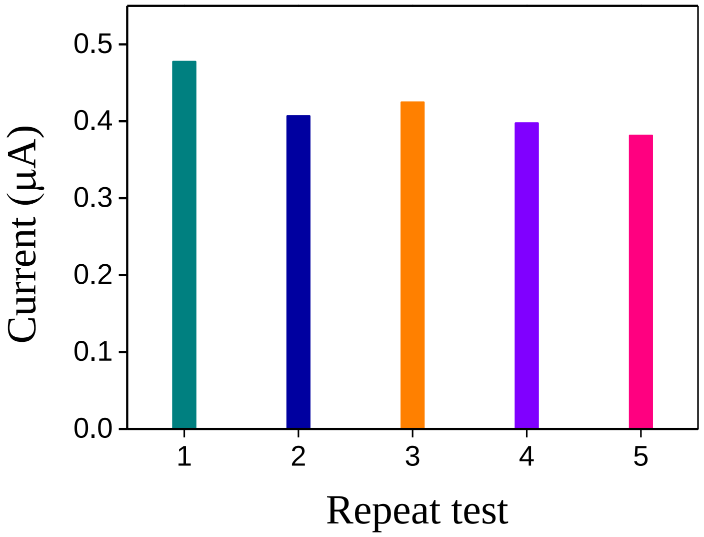

Figure S11. Electrochemical stability of I-Phene toward NO sensing.

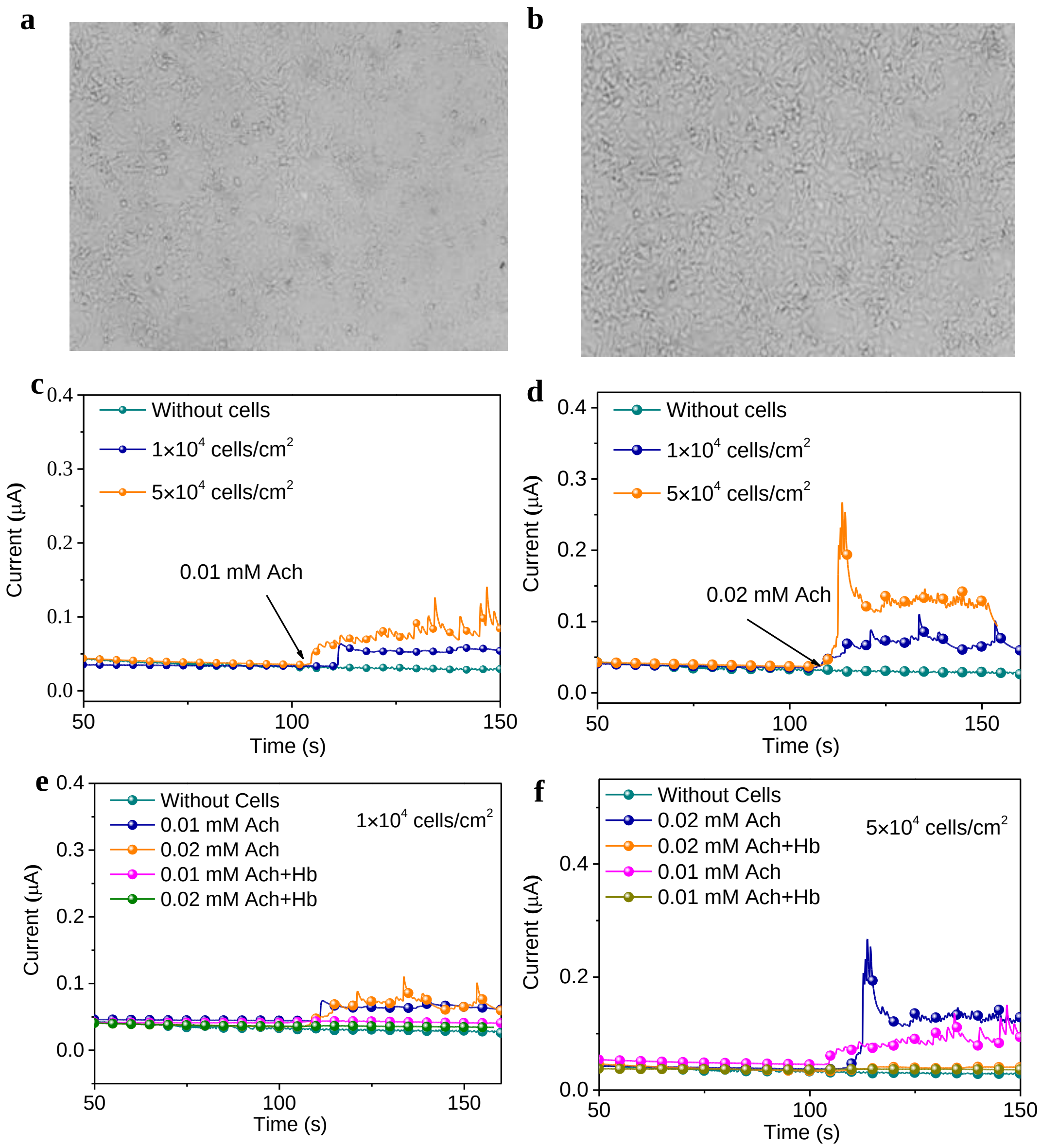

Figure S12. Pictures of different concentrations of Hela cells a) 1×10^4 cells/cm^2 and b) 5×10^4 cells/cm^2. I-Phene Stimulated by c) 0.01 mM and d) 0.02 mM of Ach, e) 1×10^4 cells/cm^2 and f) 5×10^4 cells/cm^2.

References

[1] C. M. Li, J. Zang, D. Zhan, W. Chen, C. Q. Sun, A. L. Teo, Y. T. Chua, V. S. Lee, S. M. Moochhala, *Electroanalysis*, **2006**, *18*, 713.

[2] Y. Zhang, S. Y. Lu, Z. Shi, Z. L. Zhao, Q. Liu, J. C. Gao, T. Liang, Z. Zou, C. M. Li, *Nanoscale*, **2019**, *11*, 4471.

[3] H. Xu, C. Liao, Y. Liu, B. C. Ye, B. Liu, *Anal. Chem.*, **2018**, *90*, 4438.

[4] X. Zhao, K. Wang, B. Li, C. Wang, Y. Ding, C. Li, L. Mao, Y. Lin, *Anal. Chem.*, **2018**, *90*, 7158.

[5] J. Li, J. Xie, L. Gao, C. M. Li, *ACS Appl. Mater. Inter.*, **2015**, *7*, 2726.

[6] R. Geetha Bai, K. Muthoosamy, M. Zhou, M. Ashokkumar, N. M. Huang, S. Manickam, *Biosens. Bioelectron.*, **2017**, *87*, 622.

[7] N. I. Ikhsan, P. Rameshkumar, N. M. Huang, *RSC Adv.*, **2016**, *6*, 107141.

[8] M. M. Shahid, P. Rameshkumar, A. Pandikumar, H. N. Lim, Y. H. Ng, N. M. Huang, *J. Mater. Chem. A*, **2015**, *3*, 14458.

[9] F. X. Hu, J. L. Xie, S. J. Bao, L. Yu, C. M. Li, *Biosens. Bioelectron.*, **2015**, *70*, 310.
